# UHPLC-UV/Vis Quantitative Analysis of Hydroxylated and *O*-prenylated Coumarins in Pomegranate Seed Extracts

**DOI:** 10.3390/molecules24101963

**Published:** 2019-05-22

**Authors:** Serena Fiorito, Federica Ianni, Francesca Preziuso, Francesco Epifano, Luca Scotti, Tonino Bucciarelli, Salvatore Genovese

**Affiliations:** 1 Department of Pharmacy, University “G. d’Annunzio” of Chieti-Pescara, Via dei Vestini 31, 66100 Chieti Scalo (CH), Italy; serena.fiorito@unich.it (S.F.); francesca.preziuso@unich.it (F.P.); s.genovese@unich.it (S.G.); 2 Department of Pharmaceutical Sciences, University of Perugia, Via Fabretti 48, 06123 Perugia, Italy; federica.ianni@chimfarm.unipg.it; 3 Department of Oral, Medical, and Biotechnological Sciences, University “G. d’Annunzio” of Chieti-Pescara, Via dei Vestini 31, 66100 Chieti Scalo (CH), Italy; luca.scotti@unich.it (L.S.); tonino.bucciarelli@unich.it (T.B.)

**Keywords:** Auraptene, Lythraceae, nutraceuticals, oxyprenylated coumarins, *Punica granatum*, UHPLC-UV-Vis, umbelliprenin

## Abstract

A simple and rapid analytical UHPLC methodology with spectrophotometric (UV/Vis) detection, coupled with different extraction procedures, has been perfected to investigate the presence of biologically active *O*-prenylated umbelliferone derivatives, such as auraptene and umbelliprenin, in pomegranate (*Punica granatum* L.) seed extracts. Absolute ethanol was the most efficient extraction solvent in terms of yields, after a short ultrasound-assisted. The highest concentration values recorded under these experimental conditions were 1.99 μg/g of dry extract and 6.53 μg/g for auraptene and umbelliprenin, respectively. The parent metabolite umbelliferone was also detected (0.67 μg/g). The extraction and UHPLC analytical methodology set up in the present study proved to be an efficient, powerful, and versatile technique for the simultaneous qualitative analysis and quantification of oxyprenylated coumarins in pomegranate seed extracts. The characterization of such secondary metabolites in the mentioned phytopreparation represents, to the best of our knowledge, the first example in the literature.

## 1. Introduction

**Figure 1:**
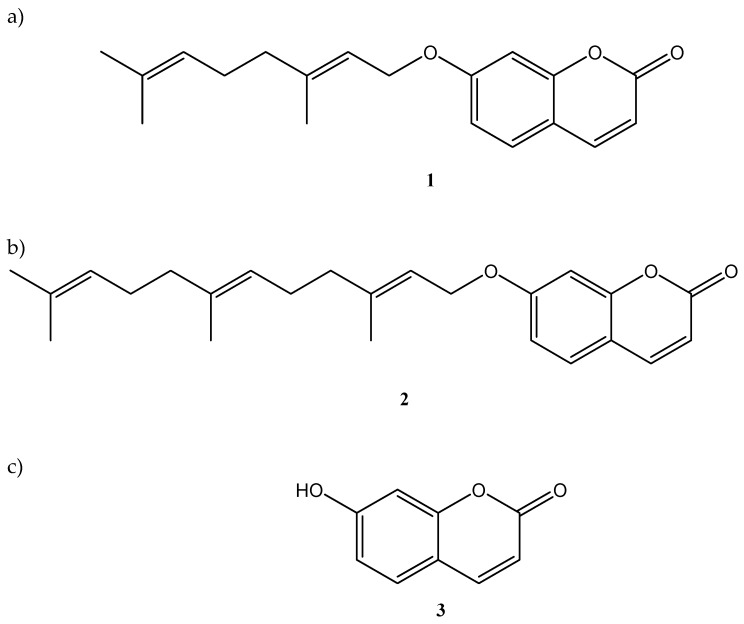
Chemical structures of (**a**) auraptene **1**, (**b**) umbelliprenin **2**, and (**c**) umbelliferone **3**.

Pomegranate (*Punica granatum* L., Lythraceae) is nowadays among the most fashionable and widely consumed vegetable foods worldwide. This plant has been known for at least 5000 years, and in ancient times was considered a symbol of fertility and prosperity. Due to its well documented health benefits for humans, pomegranate is used as a functional food and dietary supplement in different forms (fresh, juice, powder extract as part of pharmaceutical preparations like tablets, capsules, tea, jam, jelly, etc.) [1]. Also used as a medicinal plant, pomegranate has proven to be effective in the treatment of a wide array of health issues, such as malaria [2], dysentery [3], diabetes [4], bleeding and healing ulcers [5], gastro-intestinal disorders [6], and several others [1]. Such effects have been typically ascribed to the main phytochemicals extracted from fruits and other parts of this plant, such as anthocyanins, flavonoids, hydrolysable tannins (ellagitannins and gallotannins), lignans, triterpenes, phytosterols, alkaloids, indolamines, and coumaric and cinnamic acid derivatives [1]. For all these compounds, as part of pomegranate extracts, a plethora of literature data is available, while much less attention has been devoted to the less abundant secondary metabolites. In recent times, we have demonstrated how a class of rare, naturally occurring products, namely oxyprenylated coumarins such as auraptene **1** and umbelliprenin **2** (Figure 1a,b) are able to exert valuable and promising pharmacological activities as neuroprotective, anti-cancer, and anti-inflammatory agents [6,7,8], and to act as effective modulators of lipid and sugar metabolism, being ligands of key receptors such as the farnesoid X receptor (FXR) [9], glucose transporter type 4 (GLUT 4) [10], and peroxisome proliferator-activated receptors (PPARs) [11].

Pomegranate dietary consumption has been associated with significant improvements of metabolic syndromes, and of disorders of lipid and sugar metabolism in general [12]. Very recently, some of the targets of pomegranate phytopreparations at a biomolecular level have also been identified. These include some of the receptors triggered by auraptene **1** and umbelliprenin **2**, such as GLUT4 [13] and PPARs [14]. Recent literature data demonstrate that some of the effects associated with pomegranate consumption are a result of a synergy between its components. For example, it has been found that ellagitannins, caffeic acid, luteolin, and punicic acid from *P. granatum* seed extracts exhibit a synergistic interaction in suppressing the invasion of prostate cancer cells [15]. Thus, it can also be hypothesized that the modulatory effects on overall metabolism in humans may be the result of a synergy between all pomegranate phytochemicals, including minor ones. In this short communication, we wish to report the qualitative and quantitative analysis of auraptene **1** and umbelliprenin **2** as additional components of the phytochemical pool of pomegranate seeds. Their presence in this plant is disclosed herein for the first time. Our experimental hypothesis is also supported by the recent discoveries of hydroxylated coumarins, biosynthetic precursors of oxyprenylated ones, as minor compounds in pomegranate ethanolic seed extracts [16], and by the discovery that prenylation of phenylpropanoid cores is a biosynthetic step also typical of plants belonging to the Lythraceae family, although not so well characterized to date [17]. Moreover, the biological activities of coumarins, up to now reported to be components of the phytochemical pools of species belonging to this family, have not been detailed. 

## 2. Results and Discussion

The present study was conceived to gain further insights into phytochemical composition (in terms of oxyprenylated coumarins) of pomegranate seed EtOH extracts. Such a hypothesis was based on preliminary literature data highlighting how prenylation of phenylpropanoid cores can be effectively regarded as a part of the secondary metabolic pathways of plants belonging to the Lythraceae family. Three extraction methodologies, “classic” maceration, and ultrasound- and microwave-assisted extractions, with only absolute EtOH as the solvent, were used. This choice was primarily informed by previous results indicating that only this latter solvent performed well in terms of yields for the extraction of *O*-monoterpenyl- and *O*-sesquiterpenylcoumarins from vegetable matrices. Although hypothetically more efficient solvents are available and likely able to perform extraction with better yields, such as chloroform and/or dichloromethane, their use was not considered in this investigation, due to their toxicity for operators. Furthermore, EtOH is the only solvent other than water allowed for use in food and medicinal plant extractions by the majority of national and international pharmacopoeias. 

### 2.1. UHPLC Analysis

System suitability was estimated by analyses (10 replicates) of pure auraptene, umbelliprenin, and umbelliferone at a concentration of 5.0 μg/mL. As outlined in Table 1, retention times, retention factors, theoretical plate numbers, and peak asymmetries were within acceptable values. 

Calibration curves were drawn in the concentration range 0.5–50 μg/mL for all standards, and were linear over this range. Determination coefficients (*r^2^*) were >0.9986, as reported in Table 2. 

A linear regression analysis with weighting factors equal to 1/x^2^ was employed to determine the relationship between concentration of chemical standards auraptene **1**, umbelliprenin **2**, and umbelliferone **3**, and the detector response. Limit of quantification (LOQ) of the method was defined as expected by the International Guidelines ICH Q2 (R1). Limit of detection (LOD) and LOQ were calculated using results obtained from chromatographic runs of three replicates of real samples spiked at different concentrations (QC_Low_ = 2.5 μg/mL, QC_Medium_ = 25 μg/mL, and QC_High_ = 45 μg/mL, signal-to-noise ratio of 3 and 10, respectively). Both values were the same for the three analytes (0.5 μg/mL). Data for intra- and inter-day precision and accuracy were obtained from the analysis of QC samples at three different concentrations (QC_Low_, QC_Medium_, and QC_High_) in duplicate on the same day and for five consecutive days, as reported in Table 3.

RSD values for intra- and inter-day precision did not exceed 5.9%, while the corresponding values for accuracy (BIAS%) ranged from −3.4% to 3.25%. All peaks were resolved at the baseline and no appreciable carry over effects were recorded. Although, we recorded shorter retention times for the same phytochemicals in our recently published papers about very similar topics [18,19], in the case of pomegranate seeds, we had to apply a more complicated gradient elution profile to achieve sharper profiles of peaks of the three analytes under investigation. All other attempts to reduce retention times by modification of the mobile phase composition resulted in an altered shape of the chromatographic peak, preventing its exact quantification. Recoveries of auraptene **1**, umbelliprenin **2**, and umbelliferone **3** were >96.4% with a good precision (RSD < 3.0%).

### 2.2. Quantification of Auraptene, Umbelliprenin, and Umbelliferone in Pomegranate Seed Extracts

**Figure 2:**
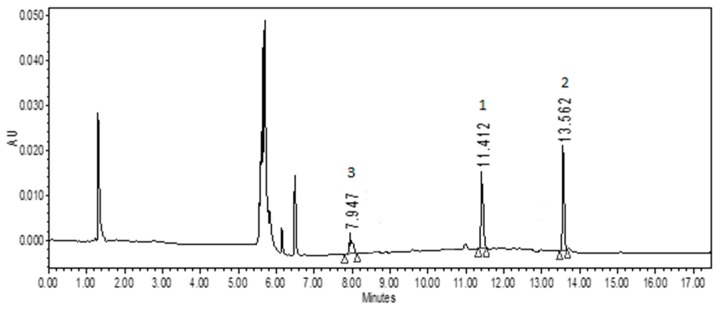
UHPLC chromatograms of pure auraptene **1**, umbelliprenin **2**, and umbelliferone **3** in pomegranate seeds ethanolic extract (ultrasound-assisted maceration, 1 min.).

In this study we used three methodologies to obtain pomegranate seed ethanolic extracts: overnight maceration and ultrasound- and microwave-assisted extractions for periods of time ranging from 1 to 10 min. The ultrasound-assisted maceration, accomplished over a period of 1 min, performed significantly better in terms of yields (around 5- to 18-fold) than all other methodologies (data not shown). Results of the quantification, adopting this last set of experimental conditions, were as follows: 1.99 ± 0.08 μg/g of dry extract for auraptene **1**, 6.53 ± 0.12 μg/g umbelliprenin **2**, and 0.67 ± 0.04 μg/g for umbelliferone **3** (values expressed as means ± standard deviation (*n* = 3)). Retention times of auraptene **1**, umbelliprenin **2**, and umbelliferone **3** resulting from analysis of the vegetable matrix matched those recorded from the chromatographic runs accomplished with pure reference compounds. Chromatograms of the extract are shown in Figure 2. Furthermore, MS spectra recorded for auraptene, umbelliprenin, and umbelliferone from pomegranate seed extracts exactly match those obtained for pure compounds deriving from chemical synthesis and already described [18,19].

The initial hypothesis, based on recent discoveries about very preliminary characterization of prenylated secondary metabolites in some species belonging to the Lythraceae family, was verified by the detection of auraptene **1** and umbelliprenin **2** as components of pomegranate seeds. Their biosynthetic precursor, umbelliferone **3**, was also recorded in very low amounts. Quite surprisingly, no phytochemicals with shorter *O*-side chains (e.g., 7-isopentenyloxycoumarin) were detected, allowing us to hypothesize that the biosynthesis of secondary metabolites of mixed origin in *P. granatum*, like oxyprenylated coumarins, is limited to transfer to phenylpropanoid cores of only monoterpenyl and sesquiterpenyl skeletons. Findings described herein give new insights into the chemical composition of pomegranate seed extracts, representing one of the most consumed worldwide food supplements nowadays, and indicate how auraptene **1** and umbelliprenin **2** can be considered additional bioactive principles of the phytochemical pool reported to date for *P. granatum*. The characterization of these two secondary metabolites also represents an enforcement of the nutritional and nutraceutical value of pomegranate and its food and phytopreparation, and allows us to hypothesize their active role (as a part of a phytocomplex in synergy with major chemicals like ellagic acid, tannins, and flavonoids) underlying the observed healthful and therapeutic effects of *P. granatum*. Quantification and qualitative characterization of oxyprenylated umbelliferone derivatives **1** and **2**, as detailed herein, may also be helpful to address further studies to better define the prenylated phytochemical profiles of *P. granatum* and chemotaxonomically related species.

The methods described herein represent a slight variation of those applied for our recently reported investigations aimed at characterizing the presence of rare oxyprenylated secondary metabolites in medicinal and food plants [20,21]. Comparing results recorded herein with those obtained with previous applications of the same UHPLC methodology, it is evident that the latter was demonstrated to be particularly suitable for the qualitative and quantitative analysis of oxyprenylated phenylpropanoids, leading to very good results, despite very different plant extraction methods and overall chemical composition of matrices (e.g., deep eutectic solvent dispersive liquid–liquid micro-extraction from vegetable oils [20], solid-phase extraction from essential oils [21], and “classic” use of EtOH for a matrix rich in polyphenols, as depicted herein).

## 3. Materials and Methods 

### 3.1. Chemicals, Plant Materials, and Extraction Procedures 

Umbelliferone **3** (purity > 99%), was purchased from Merck Sigma-Aldrich (St. Louis, MI, USA). Auraptene **1** and umbelliprenin **2** were obtained by chemical synthesis following the previously reported procedure [7]. Their purity was >98.3% (HPLC and ^1^H NMR) [7]. Acetonitrile and formic acid were purchased from DasitGroup-Carlo Erba Reagenti (Milan, Italy). H_2_O was obtained using a Milli-Q Ultrapure purification system (Millipore, Bedford, MA, USA). Pomegranate fruits (geographical origin: Sicily, Italy) were purchased from a local market. A voucher specimen of seeds, named PG-S-001, has been kept in the deposit of the laboratory of Chemistry of Natural Compounds at the Department of Pharmacy of the University of Chieti-Pescara. Seeds were collected from fresh fruits and homogenized prior to extractions. A total 500 mg of vegetable material was extracted with 5 mL of absolute EtOH using three different methods: “classic” maceration (24 h), and ultrasound- and microwave-assisted extraction (1 to 10 min), using methods very similar to those already reported in the literature for previous studies of oxyprenylated metabolites [22]. Resulting solutions were centrifugated at 13,000 rpm (5 min), filtered on a 0.22 μm PTFE filter, and concentrated to a total volume of 0.5 mL for each sample to be analyzed. Finally, a volume of 5 μL was injected into UHPLC. 

### 3.2. UHPLC Analysis

A total 10 mg each of auraptene **1** and umbelliprenin **2** (1.0 mg/mL) were dissolved into 10 mL of MeOH to obtain separate stock solutions, which were subsequently combined and stored in amber vials at 1–3 °C before analysis. The UHPLC apparatus consisted of a Waters Ultra Performance Liquid Chromatography system (ACQUITY H-Class) equipped with an Acquity UHPLC^®^ BEH C_18_ (50 × 2.1 mm I.D. 1.7 μm particle size) column thermostat at 25 ± 1 °C, and protected by a disposable Security Guard C_18_ (4.6 × 2.1 mm I.D.) (Phenomenex, Torrance, CA, USA). Mobile phase consisted of H_2_O/CH_3_CN (90/10) containing 0.04% of formic acid (A), and CH_3_CN containing 0.04% of formic acid (B) in gradient elution, as follows: 100% A at a flow rate of 0.2 mL/min from 0 to 4.1 min, 50% A and 50% B at a flow rate of 0.3 mL/min from 4.1 to 12 min, 100% B at a flow rate of 0.3 mL/min from 12.1 to 18 min, 100% A at a flow rate of 0.2 mL/min from 18.1 to 25 min. UV detection wavelength was 320 nm. Working solutions of the two standards at concentrations of 0.5, 1, 2.5, 5, 10, 20, 25, 30, 40, 45, and 50 μg/mL were obtained by dilution of stock ones with the mobile phase, and used for processing of calibration curves. These were plotted using weighted linear least-squares regression analysis according to the equation y = a + bx (“y” = peak area of analyte, “x” = concentration of the analyte in the samples, “a” = intercept, “b” = slope of the regression line). Concentration values of quality control (QC) and samples to be quantified were recorded by the interpolation of analyte peak areas on the calibration curve. Empower v.3 software (Waters) was used for the overall management of UHPLC analysis. The method was validated for the following parameters: linearity (calculated in the range 1–50 μg/mL for each analyte), limit of detection (LOD) and limit of quantification (LOQ), accuracy, and precision as expected by the ICH guidelines. Precision was recorded at three concentration levels (QC_Low_ = 2.5 μg/mL, QC_Medium_ = 25 μg/mL, and QC_High_ = 45 μg/mL) in three replicates, providing the RSD of the determination. Accuracy was determined using pomegranate seed extract samples spiked in triplicates with standard solution at three concentration levels (QC_Low_, QC_Medium,_ and QC_High_). Three chromatographic runs were accomplished for each determination. A recovery test was also accomplished to evaluate the accuracy. Certain amounts of the three pure chemical standards were added to a pomegranate seed extract sample and analyzed following the same experimental conditions as above. Recovery of each analyte was calculated according to the method of Wand and coworkers [23]. Selectivity was established by the comparison of calibration curves of auraptene and umbelliprenin in MeOH with standard calibration curves of pomegranate seed extract containing the lowest concentration of the selected reference compound. 

## 4. Conclusions

The investigation described herein is a part of an ongoing series of studies devoted to characterizing minor phytochemicals in widely consumed food plants claimed to exert beneficial effects for human welfare. In this context, we applied an already reported easy and effective extractive procedure and UHPLC methodology for the qualitative and quantitative analysis of two *O*-prenyl metabolites. Our efforts provided the characterization for the first time not only in *P. granatum*, but in the overall Lythraceae family, of two 7-oxyprenylated coumarins, auraptene **1** and umbelliprenin **2**. The present study may be a great stimulus to address further research activities aimed at recording the presence of minor biologically active components in food and medicinal plants, especially considering the Lythraceae family comprises some other edible vegetables like *Trapa bicornis* L., commonly known as “water caltrop,” and at least four *Sonneratia* species, namely *Sonneratia alba* Sm., *S. caseolaris* (L.) Engl., locally known as “crabapple mangrove,” *S. hainanensis* W.C.Ko, E.Y.Chen & W.Y.Chen, and *S. ovata* Backer.

## Figures and Tables

**Table 1:** Parameters of the system suitability of the UHPLC methodology.

Compound	1	2	3
Retention time (min) ^a,b^	11.41 (0.4%)	13.56 (0.4%)	7.94 (0.3%)
Peak symmetry (10%)	1.05	1.08	1.17

**^a^** Values in brackets represent RSD % of the retention time (10 replicates), **^b^** T_0_ was calculated uracil eluted adopting the same experimental conditions (t_0_ = 1.46).

**Table 2:** Slope, intercept, r^2^, and recovery of the two UHPLC standard.

Compound	Slope	Intercept	r^2^	Recovery (%)
1	181,298 (167,944–194,709)	25,055 (−145,287–195,411)	0.9991	99.8
2	46,555 (42,578–50,521)	10,802 (−39,690–61,300)	0.9986	100.3
3	23,565 (21,967–25,163)	7985 (−18,712–34,520)	0.9989	96.4

**Table 3:** Precision and accuracy data.

Compound	1	2	3
Precision			
Intra-day (*n = 6*)	3.7–6.7	1.5–4.1	2.3–4.8
Inter-day (*n = 6*)	4.3–7.6	0.8–5.4	1.2–3.1
Accuracy			
Intra-day (*n = 6*)	1.4–3.4	0.8–2.7	1.5–3.6
Inter-day (*n = 6*)	1.5–3.6	1.3–2.6	1.1–2.9
